# Metformin potentiates arsenic disulfide in diffuse large B-cell lymphoma: modulation of BAX/BCL-2 apoptotic pathway and hedgehog pathway

**DOI:** 10.3389/fonc.2026.1933274

**Published:** 2026-09-01

**Authors:** Jiahao Zhang, Huawei Li, Mingxin Zhao, Chen Chen, Shenghong Du, Ling Wang

**Affiliations:** 1 Qingdao Medical College of Qingdao University, Qingdao, Shandong, China; 2 Department of Hematology, The Affiliated Tai’an City Central Hospital of Qingdao University, Tai’an, Shandong, China; 3 Department of Information, The Affiliated Tai’an City Central Hospital of Qingdao University, Tai’an, Shandong, China

**Keywords:** apoptosis, arsenic disulfide, diffuse large B-cell lymphoma, hedgehog signaling, metformin

## Abstract

**Background:**

Arsenic disulfide (As_2_S_2_) inhibits proliferation and induces apoptosis in diffuse large B-cell lymphoma (DLBCL) cells, its clinical application is constrained by toxicity. Metformin has also been reported to exert antitumor effects across various malignancies. This study investigated whether metformin potentiates the anti-lymphoma efficacy of As_2_S_2_ in DLBCL cells and evaluated associated alterations in apoptosis-related markers and Hedgehog signaling molecules.

**Methods:**

DLBCL cell lines (DB and SU-DHL-4) were treated with metformin and/or As_2_S_2_. Cell viability was assessed using the Cell Counting Kit-8 (CCK-8) assay, apoptosis was determined by Annexin V/propidium iodide (PI) flow cytometry, and drug interactions were analyzed using the Bliss independence model and CompuSyn software. The expression levels of BAX, BCL-2, SMO, GLI1, and GLI2 were analyzed by quantitative real-time PCR and Western blotting.

**Results:**

Both metformin and As_2_S_2_ inhibited DLBCL cell proliferation in a dose- and time-dependent manner. Combination treatment resulted in significantly greater growth inhibition in both cell lines compared with either monotherapy, with synergistic effects confirmed by both Bliss and CompuSyn analyses. Furthermore, the combination significantly increased apoptosis relative to single-agent treatments. At the molecular level, combined treatment was associated with a pro-apoptotic shift in the BAX/BCL-2 ratio and downregulation of Hedgehog pathway components, including SMO, GLI1, and GLI2, with more pronounced changes observed at the protein level.

**Conclusions:**

Metformin enhances the anti-proliferative and pro-apoptotic effects of As_2_S_2_ in DLBCL cells *in vitro*. These effects are associated with modulation of the BAX/BCL-2 axis and suppression of Hedgehog pathway-related molecules. Further studies are warranted to elucidate the underlying mechanisms and to evaluate efficacy and safety *in vivo*.

## 1 Introduction

Diffuse large B-cell lymphoma (DLBCL) is the most prevalent subtype of adult lymphoma and presents a significant clinical challenge due to its marked biological heterogeneity (1–3). Although the introduction of anti-CD20-based immunochemotherapy has substantially improved clinical outcomes, a considerable proportion of patients develop relapsed or refractory disease. Despite recent advances in immunotherapy, including chimeric antigen receptor (CAR) T-cell therapy, many patients with relapsed/refractory DLBCL remain incurable (4, 5). Consequently, the identification of novel therapeutic vulnerabilities and the development of rational combination strategies are prioritized research objectives. Tumor metabolic adaptation has emerged as a potential therapeutic vulnerability.

Metformin, an oral biguanide widely employed as first-line therapy for type 2 diabetes mellitus, has garnered significant interest due to its reported antitumor activity across various malignancies. Clinical and epidemiological studies suggest that metformin use may be associated with reduced cancer incidence and cancer-related mortality in certain contexts (6, 7). In DLBCL, hyperglycemia and diabetes-related metabolic dysregulation are linked to adverse prognosis, whereas metformin use is associated with improved outcomes in patients with concomitant diabetes (8–10). These observations underscore the biological relevance of metabolic processes in lymphoma progression (11, 12). However, the direct antitumor efficacy of metformin monotherapy appears limited, suggesting that its clinical potential may reside primarily in mechanism-based combination strategies rather than as a standalone intervention.

As_2_S_2_ has also been reported to exert antiproliferative and pro-apoptotic effects in DLBCL cells. Previous studies, including our preliminary data, indicate that As_2_S_2_ may modulate the balance of apoptosis-related proteins by increasing the BAX/BCL-2 ratio and suppress components of the Hedgehog signaling pathway, such as SMO and GLI1 (13–15).This is particularly relevant given that aberrant Hedgehog signaling contributes to lymphoma cell survival, malignant progression, and treatment resistance (16). In canonical Hedgehog signaling, activation of Smoothened (SMO) leads to the downstream activation of GLI transcription factors, thereby promoting transcriptional programs that support tumor maintenance. Collectively, these findings suggest that both apoptotic regulation and Hedgehog signaling are pertinent to the response of DLBCL cells to As_2_S_2_. Metformin has been reported to inhibit lymphoma growth via pathways involving AMPK activation and mTOR suppression, and it has been shown to enhance the antitumor activity of arsenic trioxide in other tumor models (17–19).However, whether metformin can potentiate the activity of As_2_S_2_ in DLBCL remains unclear. Specifically, it is unknown whether any synergistic effect between these two agents is mediated by alterations in the BAX/BCL-2 apoptotic axis and Hedgehog signaling, which remain hypothetical rather than established mechanisms in this context.

To address this question, the present study evaluated the antitumor effects of metformin combined with As_2_S_2_ in DLBCL cells *in vitro*. We focused on cell proliferation, apoptosis, and treatment-associated changes in key molecules related to the BAX/BCL-2 axis and Hedgehog signaling. Our aim was to provide preliminary mechanistic evidence supporting a rational combination strategy for DLBCL and to establish a foundation for future *in vivo* and translational studies.

## 2 Materials and methods

### 2.1 Cell lines and reagents

The human DLBCL cell lines DB and SU-DHL-4 were maintained in RPMI-1640 medium supplemented with 10% fetal bovine serum (FBS) in a humidified atmosphere containing 5% CO2 at 37°C. Arsenic disulfide (As_2_S_2_; purity ≥99.53%) was obtained from Alfa Aesar, and metformin was purchased from Selleck Chemicals. Primary antibodies targeting BAX, BCL-2, SMO, GLI1, GLI2, and GAPDH were acquired from Cell Signaling Technology. Horseradish peroxidase (HRP)-conjugated goat anti-rabbit IgG secondary antibody was sourced from Zhongshan Golden Bridge. All reagents were utilized in accordance with the manufacturers’ protocols unless otherwise stated.

### 2.2 Cell proliferation assay

Cell viability was assessed using the Cell Counting Kit-8 (CCK-8) assay. DB and SU-DHL-4 cells in the logarithmic growth phase were seeded into 96-well plates at a density of 1 × 10^4^ cells per well. Following 24 h of incubation, cells were treated with metformin or As_2_S_2_ at the specified concentrations. After 24, 48, or 72 h of treatment, 10 μL of CCK-8 reagent was added to each well, and the plates were incubated for an additional 2 h. Absorbance at 450 nm was measured using a microplate reader, and cell viability was calculated relative to the untreated control group. In single-agent experiments, DB cells were exposed to As_2_S_2_ (2.5,5, 10, 20, and 40 µM) or metformin (5, 10, 20, 40, and 80 mM), while SU-DHL-4 cells were treated with As_2_S_2_ (2.5, 5,10, 20, and 40 µM) or metformin (5, 10, 20, 40, and 80 mM). The 24 h half-maximal inhibitory concentration (IC_50_) values were estimated by nonlinear regression using a four-parameter logistic model in GraphPad Prism 6.0. Based on the 24 h single-agent response profiles and preliminary CCK-8 screening, concentrations producing approximately 20%–40% growth inhibition were selected for subsequent combination experiments at submaximal, non-saturating effect levels, thereby avoiding near-maximal single-agent responses and preserving the dynamic range for evaluating drug interactions. Accordingly, DB cells were treated with 2.5, 5, or 10 mM metformin in the presence or absence of 10 µM As_2_S_2_, whereas SU-DHL-4 cells were treated with 2.5, 5, or 10 mM metformin in the presence or absence of 5 µM As_2_S_2_ for 24 h. For apoptosis, qRT-PCR, and Western blot analyses, DB cells were treated with 10 mM metformin and/or 10 µM As_2_S_2_ for 24 h, whereas SU-DHL-4 cells were treated with 5 mM metformin and/or 5 µM As_2_S_2_ for 24 h.

### 2.3 Drug interaction analysis

Drug interactions were evaluated using the Bliss independence model and CompuSyn software (version 1.0; ComboSyn Inc., Paramus, NJ, USA). According to the Bliss model, the expected additive inhibitory effect was calculated as: E_bliss = E_A + E_B - E_A × E_B, where E_A and E_B denote the fractional inhibition induced by metformin and As_2_S_2_ monotherapy, respectively. The observed combined effect was compared with the theoretical additive effect. An observed inhibition exceeding E_bliss was interpreted as synergistic. To further quantify drug interactions, the combination index (CI) was calculated using CompuSyn. A CI<1 indicates synergism, CI = 1 indicates an additive effect, and CI > 1 indicates antagonism.

### 2.4 Cell apoptosis assay

Apoptosis was quantified by flow cytometry using an Annexin V-fluorescein isothiocyanate (FITC)/propidium iodide (PI) apoptosis detection kit (KeyGen Biotech) according to the manufacturer’s protocol. After 24 h of treatment (control, metformin, As_2_S_2_, or the combination), cells were collected. Aliquots containing 1 × 10^5^ cells were incubated with Annexin V–FITC and PI for 15 min at room temperature in the dark and immediately analyzed using a flow cytometer (Becton Dickinson). Data were processed using FlowJo software (version 7.6). Cells were classified as viable (Annexin V−/PI−), early apoptotic (Annexin V+/PI−), or late apoptotic/necrotic (Annexin V+/PI+). The apoptotic fraction was defined as the sum of early and late apoptotic cells.

### 2.5 Quantitative real-time PCR assay

Total RNA was extracted from treated and untreated DLBCL cells using TRIzol reagent (Takara), and reverse transcription was performed using Takara reverse transcription reagents. Quantitative PCR amplification was conducted with SYBR Premix Ex Taq (Takara) on a Bio-Rad CFX Connect Real-Time PCR system. Gene-specific primers (Tianyi Huiyuan Biotechnology Co., Ltd.) are listed in **Table 1**. Relative mRNA expression was normalized to the housekeeping gene GAPDH and analyzed using the 2^−ΔΔCt^ method. Each gene and DNA sample was analyzed in triplicate.

**Table 1:** Primers used for the quantitative polymerase chain reaction.

Primer	Sequence (5’ to 3’)	Product, bp
H-GAPDH	Forward: ACAACTTTGGTATCGTGGAAGG Reverse: GCCATCACGCCACAGTTTC	101
H-BCL-2	Forward: GCGGATTGACATTTCTGTG Reverse: CATAAGGCAACGATCCCA	154
H-BAX	Forward: GCGACTGATGTCCCTGTCT Reverse: TGAGTGAGGCGGTGAGC	105
H-SMO	Forward: CCTGATGGCTGGTGTGG Reverse: TGAGGACAAAGGGGAGTGA	143
H-GLI1	Forward: CGCCCATGTGACCAAAC Reverse: TCTGCTTTCCTCCCTGATG	115
H-GLI2	Forward: CAAGAAGCCAAAAGTGGGA Reverse: CGCATGTCAATCGGTAGG	164

ΔCt = Ct (target gene) − Ct (GAPDH gene)

ΔΔCt = ΔCt (drug-treated cells) − ΔCt (untreated control)

### 2.6 Western blotting assay

After 24 h of treatment, total protein was extracted from DB and SU-DHL-4 cells and quantified using the BCA method. Equal amounts of protein were separated by SDS–PAGE and transferred to PVDF membranes. Membranes were blocked and incubated overnight at 4 °C with primary antibodies against BAX, BCL-2, SMO, GLI1, GLI2, and GAPDH (loading control), followed by incubation with HRP-conjugated secondary antibodies for 1 h at room temperature. Protein bands were visualized using an ECL chemiluminescence detection kit, and band intensities were quantitatively analyzed using AlphaEaseFC software.

### 2.7 Statistical analysis

Statistical analyses were performed using SPSS version 22.0. Quantitative data were expressed as mean ± standard deviation (SD). Data normality was assessed using the Shapiro-Wilk test, and homogeneity of variances was evaluated using Levene’s test. Comparisons between two groups were conducted using the independent-samples t-test or the Mann-Whitney U test, as appropriate. Comparisons among multiple groups were performed using one-way analysis of variance (ANOVA) followed by the least significant difference (LSD) *post hoc* test. Two-way ANOVA was employed for multifactorial analyses where appropriate. All statistical tests were two-sided, and a P value <0.05 was considered statistically significant. All experiments were performed in at least three independent biological replicates.

## 3 Results

### 3.1 Combined use of metformin and As_2_S_2_ synergistically inhibits DLBCL cell proliferation

**Figure 1:**
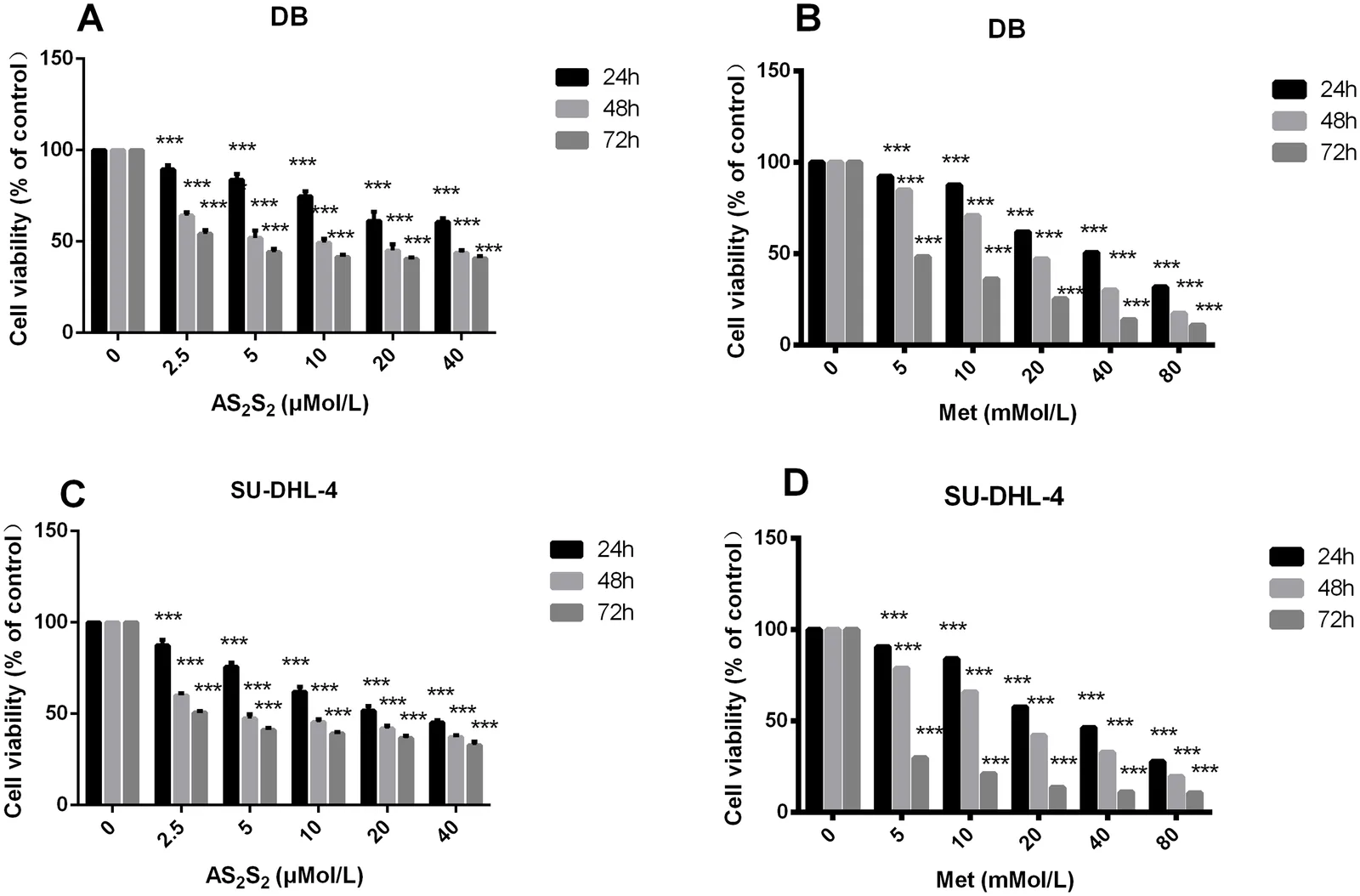
Inhibitory effects of metformin and As_2_S_2_ monotherapy on DLBCL cell proliferation assessed by CCK-8 assay (n = 6). **(A)** As_2_S_2_ at indicated concentrations in DB cells for 24, 48, and 72 h. **(B)** Metformin at indicated concentrations in DB cells for 24, 48, and 72 h. **(C)** As_2_S_2_ at indicated concentrations in SU-DHL-4 cells for 24, 48, and 72 h. **(D)** Metformin at indicated concentrations in SU-DHL-4 cells for 24, 48, and 72 h. Data are presented as mean ± SD from six independent experiments. Statistical significance was determined by two-way ANOVA followed by Bonferroni's post hoc test (***P < 0.001). (The 24 h IC_50_ values for metformin and As_2_S_2_ in DB and SU-DHL-4 cells are provided in the main text (section 3.1)).

We initially evaluated the effects of As_2_S_2_ and metformin on the viability of DLBCL cells using the CCK-8 assay. As_2_S_2_ reduced cell viability in both DB and SU-DHL-4 cells in a dose- and time-dependent manner (**Figures 1A, C**). In DB cells, treatment with 10 µM As_2_S_2_ for 24 h reduced viability to 74.67 ± 2.72% of the untreated control (P < 0.001), which further decreased to 49.30 ± 2.11% after 48 h. In SU-DHL-4 cells, 10 µM As_2_S_2_ reduced viability to 62.02 ± 2.82% at 24 h (P < 0.001) and to 45.49 ± 1.52% at 48 h. The 24 h IC_50_ values for As_2_S_2_ were 63.46 μM in DB cells and 25.09 μM in SU-DHL-4 cells. Metformin also inhibited cell proliferation in a dose- and time-dependent manner (**Figures 1B, D**). In DB cells, treatment with 5 mM metformin for 24 h reduced viability to 92.27 ± 1.35% of the control (P < 0.001).

viability to 92.27 ± 1.35% of the control (P < 0.001), decreasing to 84.78 ± 2.10% after 48 h. In SU-DHL-4 cells, viability was reduced to 90.52 ± 0.23% at 24 h (P < 0.001) and to 78.85 ± 1.50% at 48 h. The 24 h IC50 values for metformin were 37.60 mM in DB cells and 32.45 mM in SU-DHL-4 cells.

**Figure 2:**
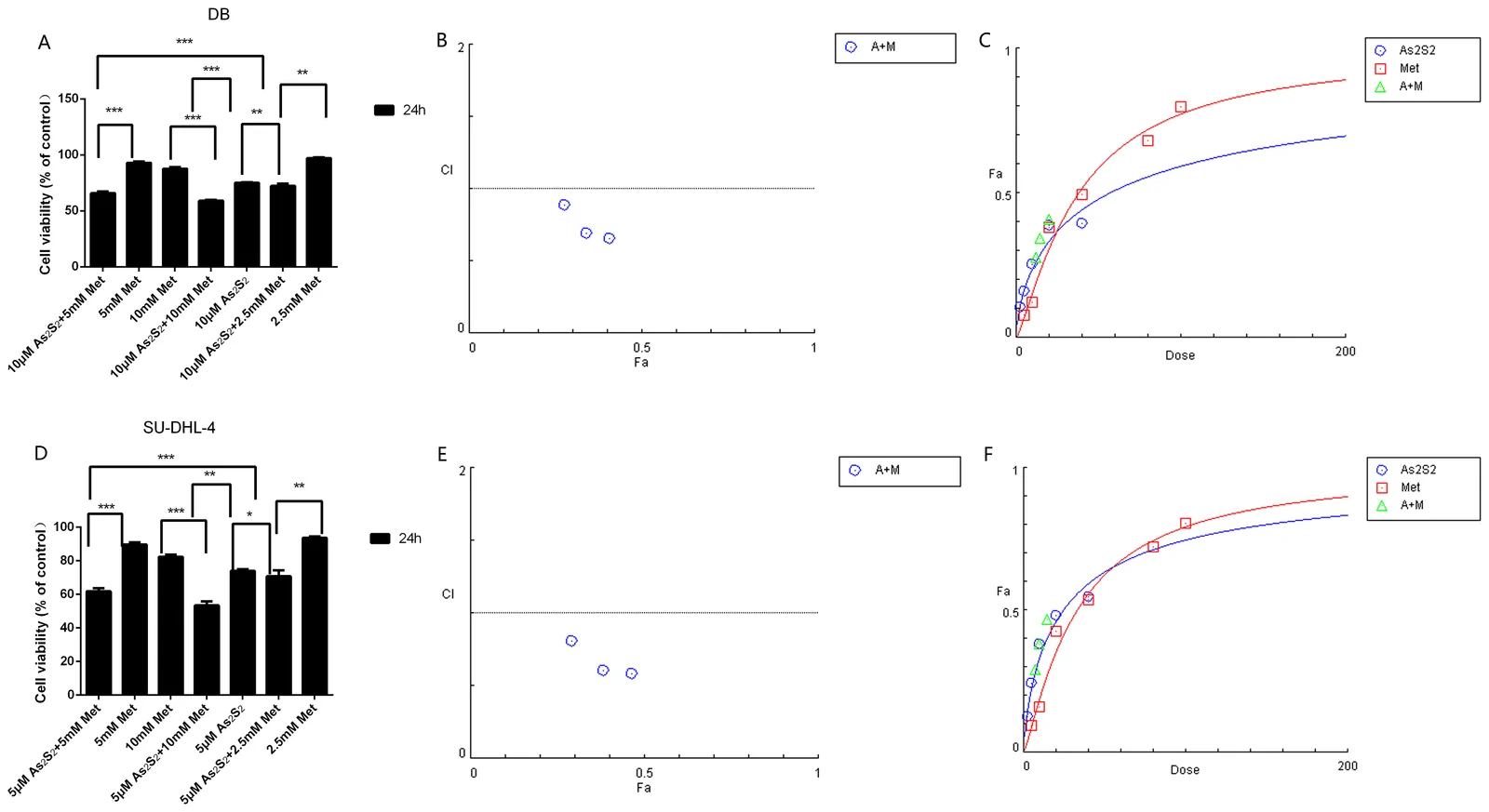
Inhibitory effects of combined metformin and As_2_S_2_ on DLBCL cell proliferation (CCK-8 assay; n = 6). **(A)** Combination versus monotherapies in DB cells at 24 h. **(B)** Combination index of metformin and As_2_S_2_ at various concentrations in DB cell: **(C)** Dose-response effects of metformin and/or As_2_S_2_ at various concentrations in DB cells; **(D)** Combination versus monotherapies in SU-DHL-4 cells at 24 h; **(E)** Combination index of metformin and As_2_S_2_ at various concentrations in SU-DHL-4 cells; **(F)** Dose-response effects of metformin and/or As_2_S_2_ at various concentrations in SU-DHL-4 cells. Data are mean ± SD from six independent experiments. Two-group comparisons were conducted with the independent-samples t test (or the Mann-Whitney U test for non-normally distributed data). Multiple-group comparisons used one-way ANOVA; and LSD-t test was performed for pairwise comparisons (*P<0.05, **P < 0.01, ***P < 0.001).

Subsequently, we investigated whether the combination of metformin and As_2_S_2_ exerted a stronger antiproliferative effect than either agent alone. At concentrations corresponding to approximately 20%–40% of the respective 24 h IC_50_ values, the combination significantly reduced viability in both cell lines compared with either monotherapy (**Figures 2A, D**). In DB cells, viability was 87.67 ± 1.43% with 10 mM metformin alone, 75.75 ± 0.01% with 10 μM As_2_S_2_ alone, and 59.11 ± 0.85% with the combination (combination vs. As_2_S_2_, P < 0.001; combination vs. metformin, P < 0.001). In SU-DHL-4 cells, viability was 89.69 ± 1.33% with 5 mM metformin alone, 73.99 ± 0.98% with 5 μM As_2_S_2_ alone, and 61.78 ± 1.97% with the combination (combination vs. As_2_S_2_, P < 0.001; combination vs. metformin, P < 0.001). Drug interaction analysis confirmed a synergistic effect. In DB cells, the expected additive inhibition calculated by the Bliss independence model was 33.59%, whereas the observed inhibition with the combination of metformin and As_2_S_2_ was 40.89%. In SU-DHL-4 cells, the expected additive inhibition was 33.64%, whereas the observed inhibition was 38.22%. In both cell lines, the observed inhibition exceeded the theoretical additive value. Consistent with these findings, CompuSyn analysis yielded CI values < 1, confirming synergism. Specifically, the combination of 10 mM metformin and 10 μM As_2_S_2_ in DB cells resulted in a CI of 0.66, and the combination of 5 mM metformin and 5 μM As_2_S_2_ in SU-DHL-4 cells resulted in a CI of 0.61 (**Figures 2B, C, E ,F**).

### 3.2 Combined use of metformin and As_2_S_2_ promotes apoptosis of DLBCL cells

**Figure 3:**
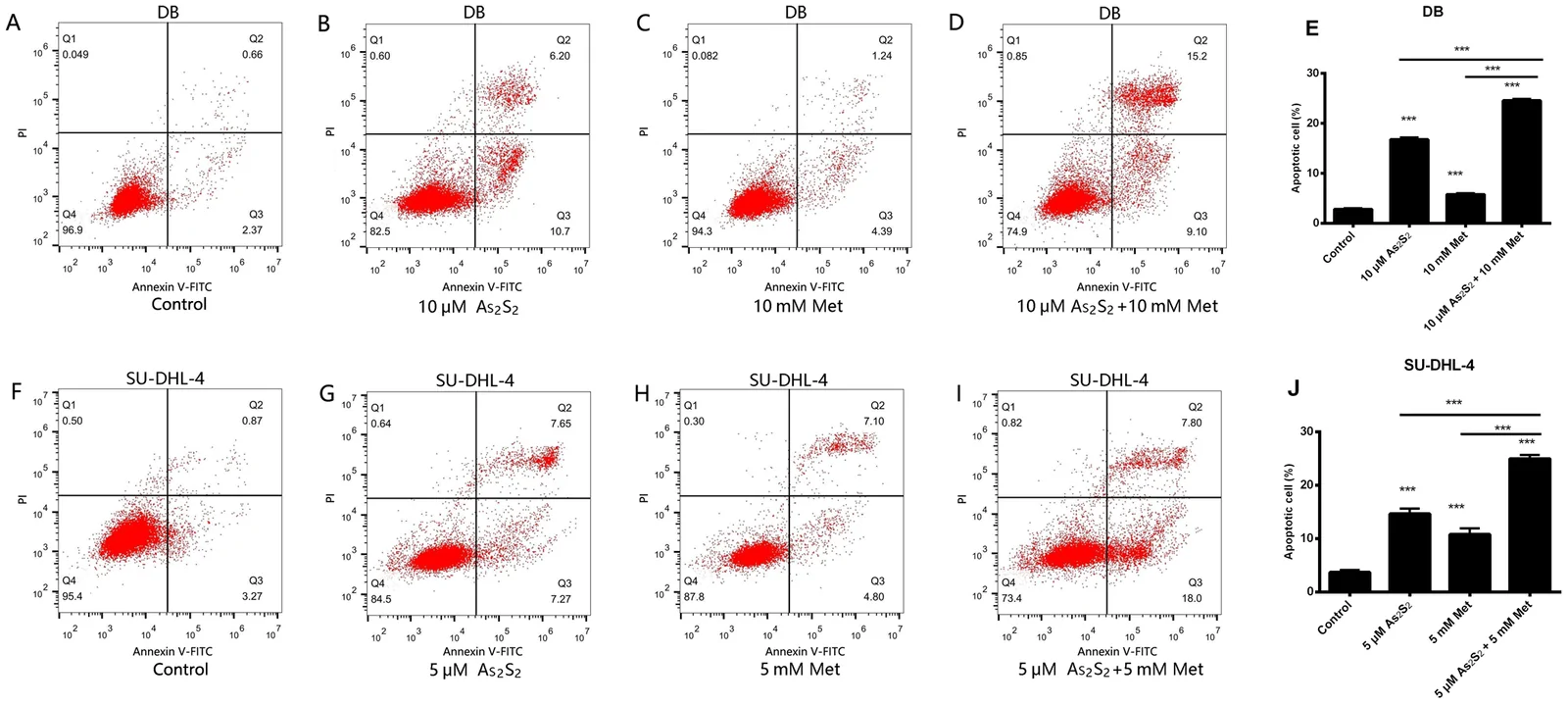
Apoptosis-indueing effects of combined metformin and As_2_S_2_ on DLBCI cells (n= 3). **(A)** Apoptotie rate of DB cells in the blank control group. **(B)** Apoptotic rate of DB cells in the As_2_S_2_: group. **(C)** Apoptotie rate of DB cells in the metformin group. **(D)** Apoptotic rate of DB cells in the metformin + As_2_S_2_ group. **(E)** Quantitative analysis of apoptotie rate in DB cells. **(F)** Apoptotic rate of SU-DHL-4 cells in the blank control group. **(G)** Apoptotie rate of SU-DHL-4 cells in the As_2_S_2_ group. **(H)** Apoptotie rate of SU-DHL-4 cells in the metformin group. **(I)** Apoptotie rate of SU-DHL-4 cells in the metformin + As_2_S_2_ group. **(J)** Quantitative analysis of apoptotie rate in SU-DHL-4 cells. Quantitative data represent total apoptotic rates (early + late apoptosis) and are presented as mean ± SD from three independent experiments. Comparisons between two groups were performed using the independent-samples t-test or the Mann–Whitney U test for non-normally distributed data. Multiple-group comparisons were condueted using one-way ANOVA followed by the LSD-t test for pairwise comparisons. (***P < 0.001).

We assessed total apoptosis (early + late) in DB and SU-DHL-4 DLBCL cells following treatment with As_2_S_2_, metformin, or their combination. In DB cells, apoptotic rates were 2.807 ± 0.236% in the control group, 16.767 ± 0.426% with As_2_S_2_ alone, and 5.760 ± 0.252% with metformin alone; notably, the combination increased apoptosis to 24.533 ± 0.321% (all P < 0.001) (**Figures 3A–E**). Similarly, in SU-DHL-4 cells, apoptotic rates were 3.677 ± 0.465% in the blank control, 14.645 ± 0.969% with As_2_S_2_ alone, and 10.797 ± 1.136% with metformin alone, whereas the combination elevated apoptosis to 24.960 ± 0.734% (all P < 0.001) (**Figures 3F–J**). These results demonstrate that co-treatment with metformin and As_2_S_2_ markedly enhances apoptotic cell death compared with either monotherapy.

### 3.3 Combined use of metformin and As_2_S_2_ regulates the expression of apoptosis- and Hedgehog pathway-related genes

**Figure 4:**
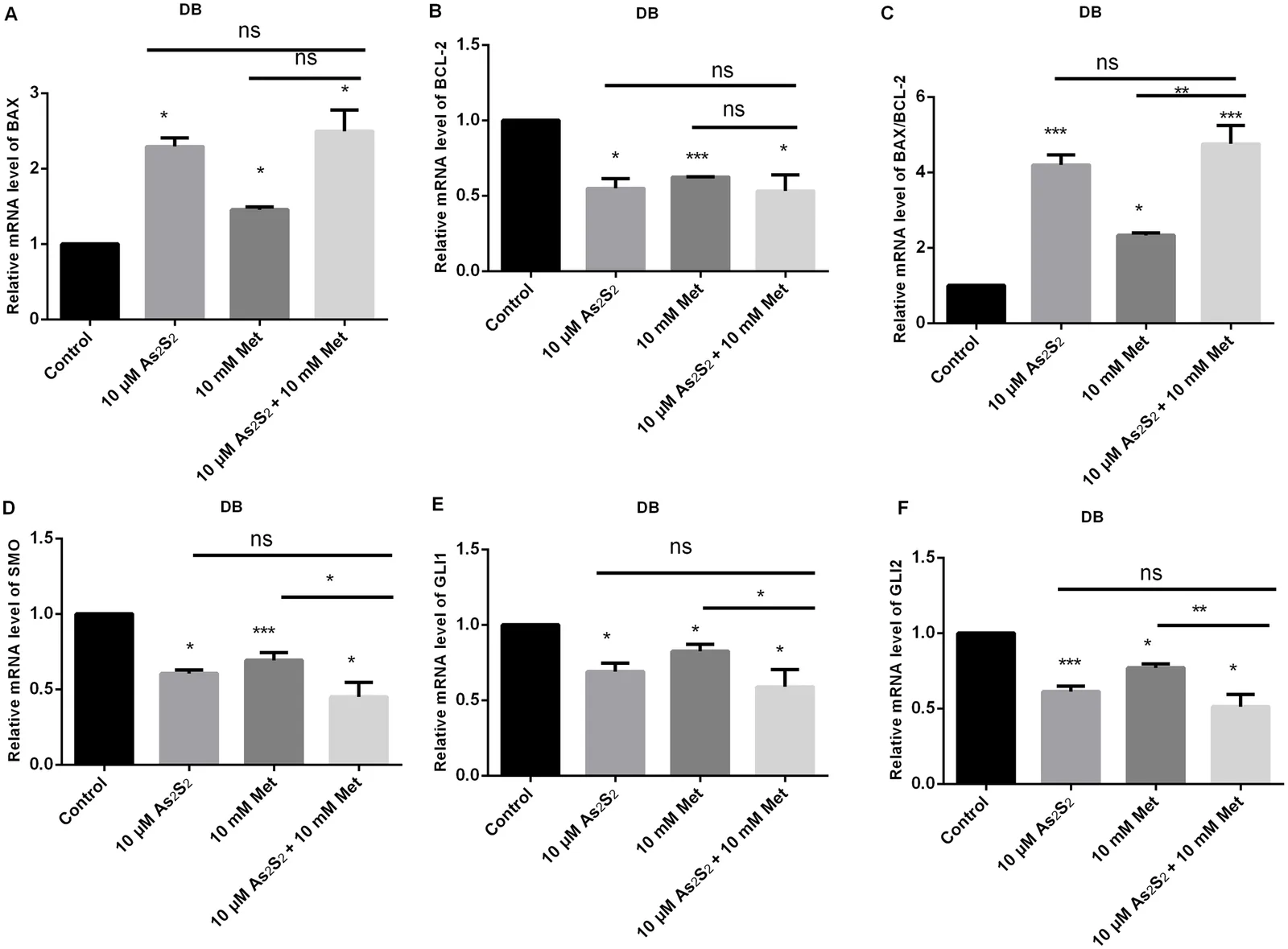
Effects of combined metformin and As_2_S_2_, on the expression of apoptosis-and Hedgehog pathway-related genes (BAX, BCL-2, SMO, GLII, and GL.I2) in DB cells (n = 3). **(A)** Quantitative analysis of BAX gene expression in DB cells. **(B)** Quantitative analysis of BCL-2 gene expression in DB cells. **(C)** Quantitative analysis of the BAX/BCL-2 expression ratio in DB cells. **(D)** Quantitative analysis of SMO gene expression in DB cells. **(E)** Quantitative analysis of GLIl gene expression in DB cells. **(F)** Quantitative analysis of GLI2 gene expression in DB cells. Gene expression levels were normalized to GAPDH and presented as mean ± SD from three independent experiments. Comparisons between two groups were performed using the independent-samples i-test or the Mann-Whitney U test for non-normally distributed data. Multiple-group comparisons were conducted using one-way ANOVA followed by the LSD-t test for painwise comparisons. (ns, not significant: *P < 0.05, **P < 0.01. ***P < 0.001).

**Figure 5:**
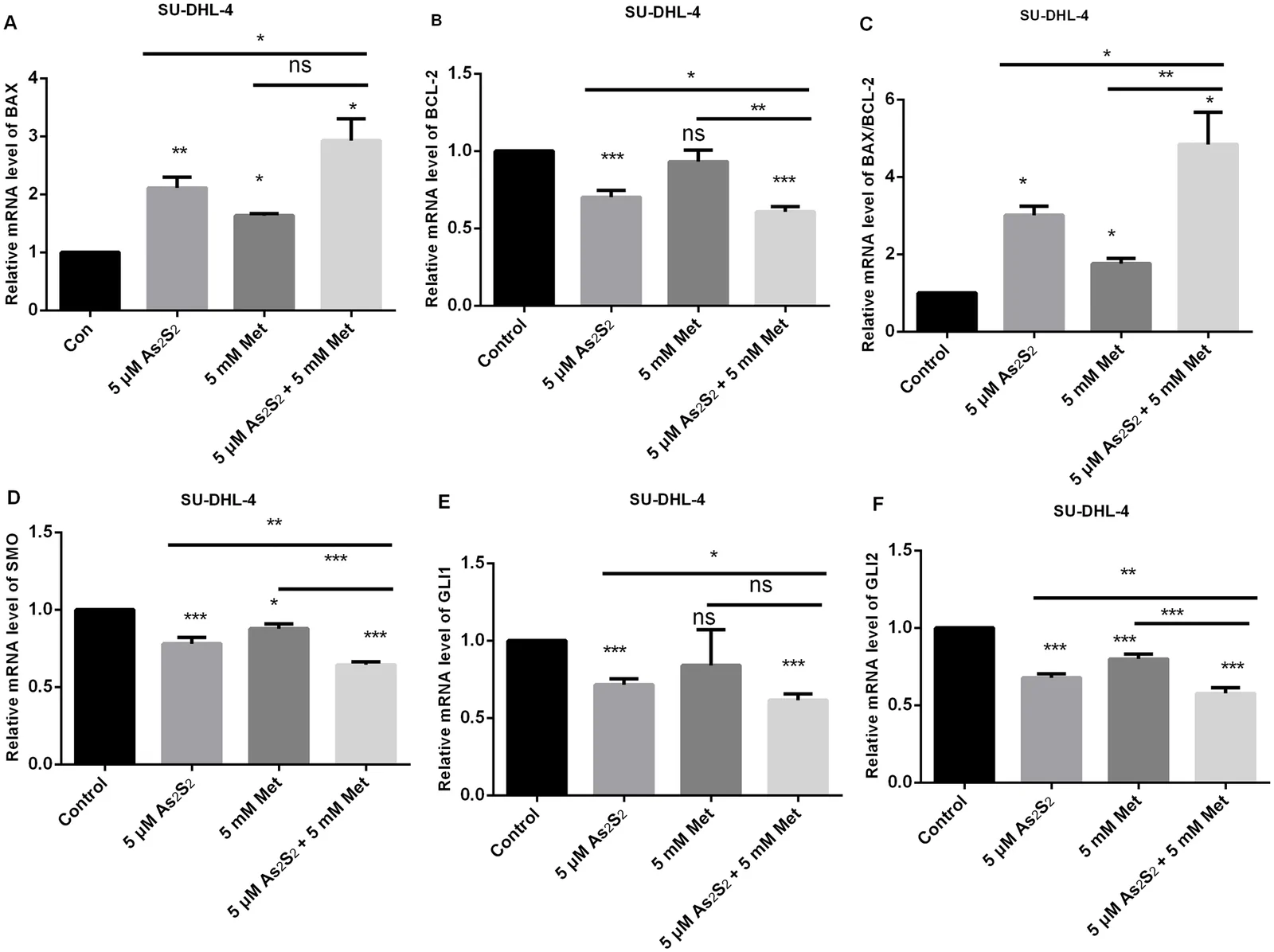
Effects of combined metformin and As_2_S_2_ on the expression of apoptosis-and. Hedgehog pathway-related genes (BAX, BCL-2, SMO, GLIT, and GLI2) in SU-DHL-4 cells (n = 3). **(A)** Quantitative analysis of BAX gene expression in SU-DHL-4 cells. **(B)** Quantitative analysis of BCL-2 gene expression in SU-DHL-4 cells. **(C)** Quantitative analysis of the BAX/BCL-2 expression ratioin in SU-DHL-4 cells. **(D)** Quantitative analysis of SMO gene expression in in SU-DHL-4 cells. **(E)** Quantitative analysis of GLIl gene expression in in SU-DHL-4 cells. **(F)** Quantitative analysis of GLI2 gene expression in in SU-DHL-4 cells. Gene expression levels were normalized to GAPDH and presented as mean ± SD from three independent experiments. Comparisons between two groups were performed using the independent-samples t-test or the Mann-Whitney U test for non-normally distributed data. Multiple-group comparisons were conducted using one-way ANOVA followed by the LSD-t test for painwise comparisons. (ns, not significant; *P < 0.05, **P < 0.01, ***P < 0.001).

We subsequently examined the mRNA expression of BAX and BCL-2 to determine whether the increased apoptosis observed with combination treatment was accompanied by alterations in the expression of apoptosis-related genes. In DB cells, metformin, As_2_S_2_, and combination treatment each increased BAX expression and decreased BCL-2 expression relative to the control group. In the combination group, BAX mRNA increased to 2.50-fold of the control, whereas BCL-2 decreased to 0.53-fold of the control (both P < 0.05 vs. control) (**Figures 4A–C**). Although the combination did not significantly differ from either monotherapy in individual BAX or BCL-2 levels, the BAX/BCL-2 ratio was higher than that observed with metformin alone, while remaining comparable to that in the As_2_S_2_ group. In SU-DHL-4 cells, combination treatment produced a more pronounced shift in apoptosis-related gene expression. BAX mRNA increased to 2.93-fold of the control, whereas BCL-2 decreased to 0.61-fold of the control (BAX: P < 0.05 vs. control and P < 0.05 vs. As_2_S_2_; BCL-2: P <0.001 vs. control, P < 0.05 vs. As_2_S_2_, and P < 0.01 vs. metformin) (**Figures 5A–C**). The combination group exhibited stronger effects than either monotherapy regarding BAX, BCL-2, and the BAX/BCL-2 ratio. Notably, metformin alone did not significantly reduce BCL-2 mRNA expression in SU-DHL-4 cells. Overall, these findings indicate that combination treatment is associated with a shift toward a pro-apoptotic BAX/BCL-2 balance, particularly in SU-DHL-4 cells.

Given that previous studies have implicated Hedgehog signaling in DLBCL cell survival, we assessed the mRNA expression of SMO, GLI1, and GLI2 following treatment. In DB cells, all treatment groups exhibited lower SMO, GLI1, and GLI2 mRNA levels than the control group. In the combination group, SMO, GLI1, and GLI2 expression decreased to 0.45-, 0.59-, and 0.51-fold of the control, respectively (SMO: P < 0.05 vs. control and P < 0.05 vs. metformin; GLI1: P < 0.05 vs. control and P < 0.05 vs. metformin; GLI2: P < 0.05 vs. control and P < 0.01 vs. metformin) (**Figures 4D–F**). Compared with metformin alone, the combination more strongly suppressed SMO and GLI2, whereas no significant difference was observed relative to As_2_S_2_ alone. GLI1 showed a similar overall trend. In SU-DHL-4 cells, combination treatment reduced SMO, GLI1, and GLI2 mRNA expression to 0.64-, 0.62-, and 0.58-fold of the control, respectively (SMO: P < 0.001 vs. control, P < 0.01 vs. As_2_S_2_, and P < 0.001 vs. metformin; GLI1: P < 0.001 vs. control and P < 0.05 vs. As_2_S_2_; GLI2: P < 0.001 vs. control, P < 0.01 vs. As_2_S_2_, and P < 0.001 vs. metformin) (**Figures 5D–F**). SMO was significantly reduced in all treatment groups compared with the control, with the greatest reduction observed in the combination group. GLI1 expression was significantly decreased by As_2_S_2_ and by the combination, whereas metformin alone did not significantly alter GLI1 expression. GLI2 was reduced in all treatment groups, with the combination exerting the strongest effect. Collectively, these results demonstrate that combination treatment is associated with reduced expression of Hedgehog pathway-related genes in both DLBCL cell lines.

### 3.4 Combined use of metformin and As_2_S_2_ regulates the expression of apoptosis- and Hedgehog pathway-related proteins

**Figure 6:**
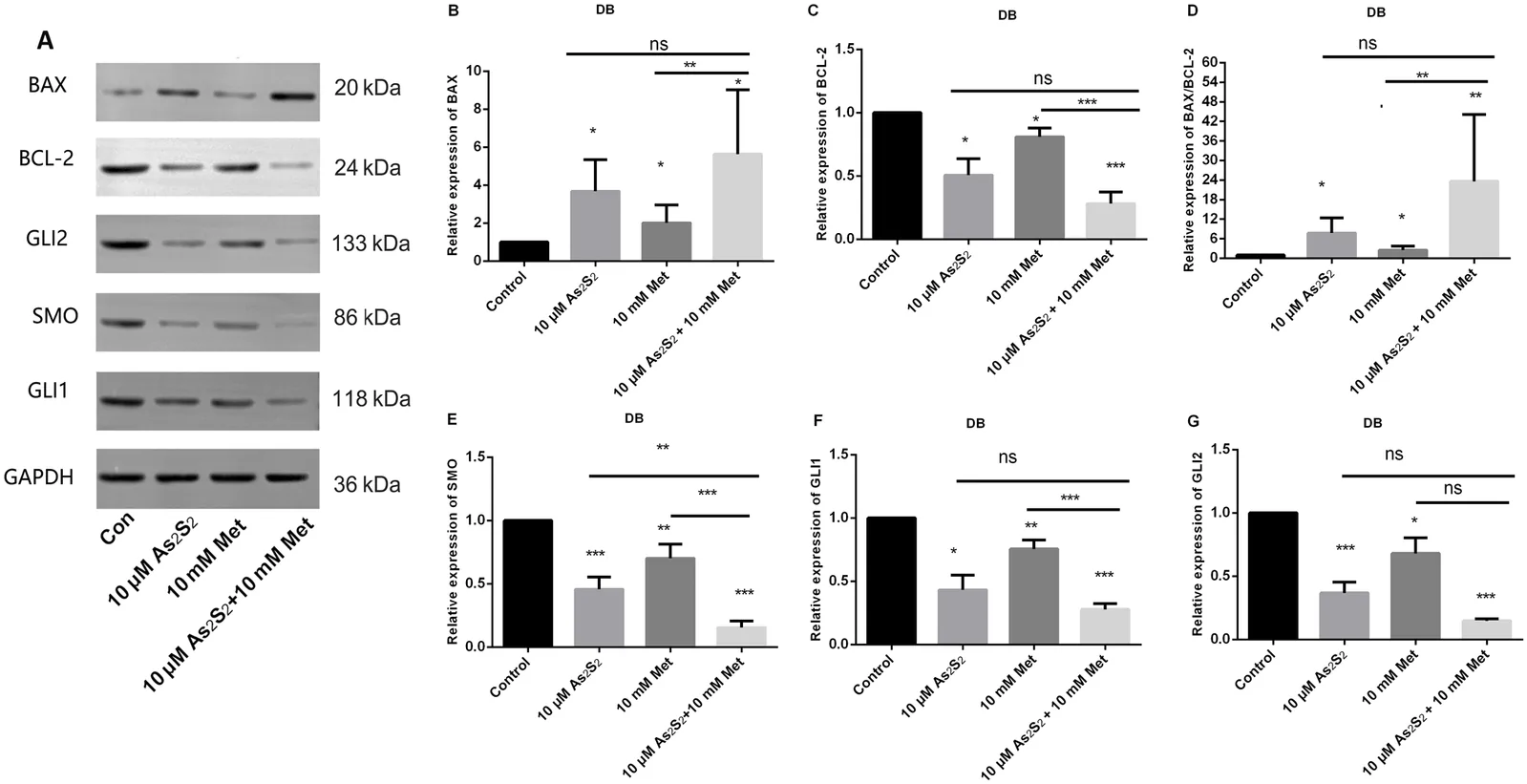
Effects of combined metformin and As_2_S_2_ on the regulation of apoptosis- and Hedgehog pathway-related proteins BAX, BCL-2, SMO, GLI1, and GL12 in DB cells (n = 3) **(A)** Western blot analysis of BAX, BCL-2, SMO, GLIl, and GLI2 protein expression. **(B)** Quantitative analysis of BAX protein expression. **(C)** Quantitative analysis of BCL-2 protein expression. **(D)** Quantitative analysis of the BAX/BCL-2 protein ratio. **(E)** Quantitative analysis of SMO protein expression. **(F)** Quantitative analysis of GLIl protein expression. **(G)** Quantitative analysis of GLI2 protein expression. Protein expression levels were normalized to GAPDH and presented as mean ± SD from three independent experiments. Comparisons between two groups were performed using the independent-samples t-test or the Mann-Whitney U test for non-normally distributed data. Multiple-group comparisons were condueted using one-way ANOVA followed by the LSD-t test for pairwise comparisons. (ns, not significant; *P < 0.05, **P < 0.01, ***P< 0.001).

**Figure 7:**
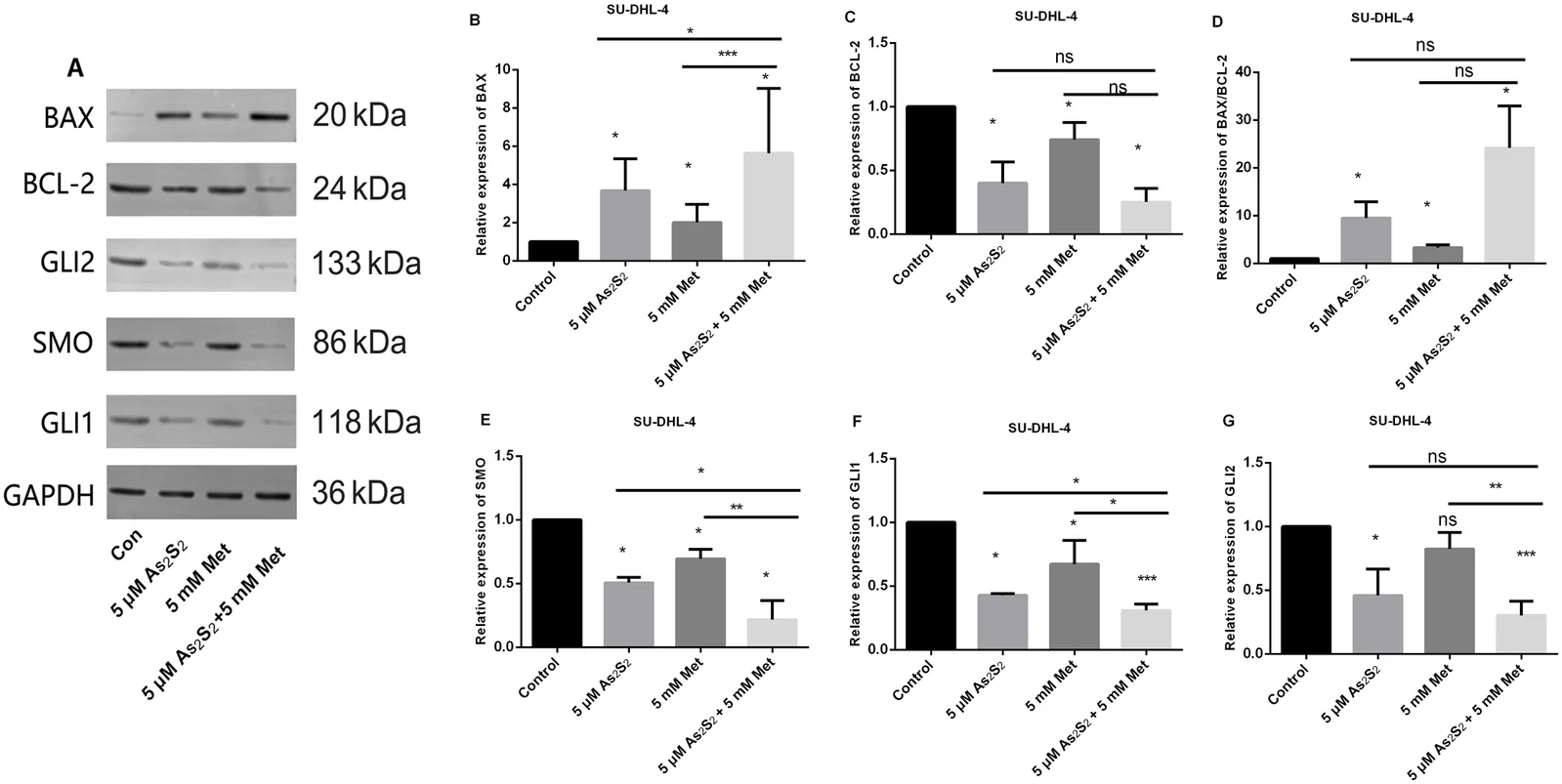
Effects of combined metformin and As_2_S_2_, on the regulation of apoptosis- and Hedgehog pathway-related proteins BAX, BCL-2, SMO, GLII, and GLI2 in SU-DHL-4 cells (n = 3). **(A)** Western blot analysis of BAX, BCL-2, SMO, GLIl, and GLI2 protein expression. **(B)** Quantitative analysis of BAX protein expression. **(C)** Quantitative analysis of BCL-2 protein expression. **(D)** Quantitative analysis of the BAX/BCL-2 protein ratio. **(E)** Quantitative analysis of SMO protein expression. **(F)** Quantitative analysis of GLIl protein expression. **(G)** Quantitative analysis of GLI2 protein expression. Protein expression levels were normalized to GAPDH and presented as mean ± SD from three independent experiments. Comparisons between two groups were performed using the independent-samples t-test or the Mann Whitney I test for non-normally distributed data. Multiple-group comparisons were conducted using one-way ANOVA followed by the LSD-t test for pairvise comparisons. (ns, not significant; *P < 0.05, **P < 0.01, ***P < 0.001).

To determine whether the transcriptional changes in apoptosis-related genes were reflected at the protein level, we measured BAX and BCL-2 expression by Western blotting. In DB cells, metformin, As_2_S_2_, and combination treatment all increased BAX protein expression and decreased BCL-2 protein expression relative to the control group. In the combination group, BAX increased to 5.64-fold of the control, whereas BCL-2 decreased to 0.28-fold of the control (BAX: P < 0.05 vs. control and P < 0.01 vs. metformin; BCL-2: P < 0.001 vs. control and P < 0.001 vs. metformin) (**Figures 6A–C**). Compared with metformin alone, the combination resulted in higher BAX and lower BCL-2 expression, whereas the corresponding values were comparable to those observed with As_2_S_2_ monotherapy. Consistent with these findings, the BAX/BCL-2 ratio was significantly increased in all treatment groups relative to the control, with a greater increase in the combination group than in the metformin group, but no significant difference relative to the As_2_S_2_ group (**Figure 6D**). A similar pattern was observed in SU-DHL-4 cells. All treatment groups significantly increased BAX and decreased BCL-2 relative to the control. In the combination group, BAX increased to 6.14-fold of the control, whereas BCL-2 decreased to 0.22-fold of the control (BAX: P < 0.05 vs. control, P < 0.05 vs. As_2_S_2_, and P < 0.001 vs. metformin; BCL-2: P < 0.05 vs. control) (**Figures 7A–D**). Compared with metformin alone, the combination produced higher BAX expression, whereas no significant differences were detected among the treated groups for BCL-2 protein or the BAX/BCL-2 ratio. These protein-level data are consistent with the apoptosis findings and support an association between combination treatment and enhanced pro-apoptotic signaling.

We then examined whether treatment affected Hedgehog pathway components at the protein level. In DB cells, metformin, As_2_S_2_, and combination treatment all reduced SMO, GLI1, and GLI2 protein expression. In the combination group, SMO, GLI1, and GLI2 were reduced to 0.15-, 0.28-, and 0.15-fold of the control, respectively (SMO: P < 0.001 vs. control, P < 0.01 vs. As_2_S_2_, and P < 0.001 vs. metformin; GLI1: P < 0.001 vs. control and P < 0.001 vs. metformin; GLI2: P < 0.001 vs. control) (**Figures 6A, E–G**). Compared with metformin alone, the combination more strongly suppressed SMO and GLI1, whereas the reduction in GLI2 was comparable among treatment groups. In SU-DHL-4 cells, combination treatment reduced SMO, GLI1, and GLI2 protein expression to 0.22-, 0.31-, and 0.30-fold of the control, respectively (SMO: P < 0.05 vs. control, P < 0.05 vs. As_2_S_2_, and P < 0.01 vs. metformin; GLI1: P < 0.001 vs. control, P < 0.05 vs. As_2_S_2_, and P < 0.05 vs. metformin; GLI2: P < 0.001 vs. control and P < 0.01 vs. metformin) (**Figures 7A, E–G**). SMO and GLI1 were decreased by all treatments, with the strongest suppression observed in the combination group. GLI2 was reduced by As_2_S_2_ and by the combination, whereas metformin alone did not significantly alter GLI2 protein expression. Taken together, these findings indicate that combination treatment is associated with the suppression of Hedgehog pathway-related proteins in both DLBCL cell lines, with more consistent effects observed at the protein level.

## 4 Discussion

In the present study, we demonstrated that metformin enhanced the anti-lymphoma activity of As_2_S_2_ in two DLBCL cell lines *in vitro*. Compared with either monotherapy, combination treatment resulted in superior growth inhibition and induced higher levels of apoptosis. These phenotypic changes were accompanied by a shift in the BAX/BCL-2 ratio toward a pro-apoptotic state and reduced expression of Hedgehog pathway-related molecules. Collectively, these findings support a cooperative antitumor interaction between metformin and As_2_S_2_ at the cellular level under the current experimental conditions.

Both metformin and As_2_S_2_ inhibited DLBCL cell proliferation in a dose- and time-dependent manner, whereas the combination treatment exerted a significantly stronger antiproliferative effect than either agent alone. One issue that warrants consideration is the relatively high concentration of metformin used *in vitro*. Consistent with previous reports, the effective concentration exceeded the plasma levels typically achievable *in vivo* (9). This discrepancy may be partly attributable to the use of RPMI 1640 medium, which contains approximately 11 mM glucose and can substantially alter cellular metabolism and treatment responsiveness (20). In this context, the use of millimolar concentrations of metformin in lymphoma models is methodologically justifiable, as prior *in vitro* studies have commonly used concentrations ranging from 4 to 16 mM, with 8–16 mM often required to induce marked apoptosis and cell-cycle arrest (9). In addition, accumulating evidence suggests that the biological effects of metformin are strongly influenced by glucose availability. Under different glucose conditions, metformin may engage distinct signaling mechanisms while maintaining an overall antiproliferative phenotype (21, 22). Therefore, glucose should be considered not merely a culture condition, but an important biological variable that shapes the cellular response to metformin. This point is particularly relevant when interpreting the translational significance of *in vitro* dose-response data.

Although As_2_S_2_ showed clear inhibitory activity as a single agent, the present data do not support it as an optimal monotherapy. Under the current experimental conditions, As_2_S_2_ monotherapy produced stronger growth inhibition than metformin monotherapy, and the combination treatment further enhanced this effect without increasing the As_2_S_2_ dose. These findings suggest that metformin may sensitize DLBCL cells to As_2_S_2_. From a translational perspective, this observation is potentially important because the clinical use of arsenical agents is often constrained by toxicity, whereas metformin has a well-established safety profile (23–25). However, because toxicity was not assessed in normal cells or *in vivo* models, no conclusion can be drawn regarding whether the combination treatment reduces arsenic-related toxicity or broadens the therapeutic window.

Mechanistically, our data indicate that apoptosis was a major contributor to the observed cooperative effect. Metformin primarily increased the BAX/BCL-2 ratio through BAX upregulation, whereas As_2_S_2_ modulated both components of this axis by increasing BAX expression and decreasing BCL-2 expression. The combination treatment produced the most pronounced shift in this balance, suggesting cooperative activation of the mitochondrial apoptotic pathway. This observation is biologically meaningful because the BAX/BCL-2 ratio is a critical determinant of mitochondrial outer membrane permeabilization and apoptotic susceptibility. An increased ratio facilitates cytochrome c release and subsequent caspase activation, thereby promoting cell death. Our findings are consistent with previous studies showing that metformin modulates apoptosis-related proteins across multiple tumor models. In ovarian cancer, metformin decreases BCL-2 and Bcl-xL levels while increasing BAX and Bad expression (26). In pancreatic cancer, metformin suppresses mTOR and STAT3 phosphorylation and reduces Mcl-1 and BCL-2 levels (27). Furthermore, in liver cancer, metformin decreases BCL-2 expression and enhances the pro-apoptotic effects of arsenicals (19). However, the upstream mechanisms underlying the observed cooperative effect remain elucidated. It is plausible that As_2_S_2_ induces stress responses, such as reactive oxygen species (ROS) generation or endoplasmic reticulum stress (28–30). While metformin augments BAX-mediated pro-apoptotic signaling through AMPK-dependent energy stress (31, 32). These possibilities are biologically plausible but require direct functional validation.

In addition to apoptosis, our findings implicate Hedgehog signaling as another potential mediator of the synergistic effect of metformin and As_2_S_2_. The Hedgehog pathway is a highly conserved signaling cascade involving ligand-receptor interactions, intracellular signal transduction, and downstream transcriptional activation; aberrant activation of this pathway has been implicated in various human malignancies (16). In DB cells, As_2_S_2_ monotherapy significantly reduced the mRNA and protein expression of SMO, GLI1, and GLI2. Furthermore, the combination treatment resulted in a greater reduction in SMO and GLI1 protein levels compared with metformin monotherapy, suggesting that As_2_S_2_ may be the primary driver of Hedgehog pathway inhibition in this cellular context. Conversely, SU-DHL-4 cells exhibited a distinct response pattern: metformin reduced GLI2 at the transcriptional level without significantly affecting GLI2 protein levels, whereas As_2_S_2_ primarily decreased GLI2 protein expression. The combination therapy suppressed GLI2 at both the transcriptional and protein levels. This pattern suggests that the two agents act at distinct regulatory layers, converging to comprehensively suppress GLI2 output. These findings align with previous studies demonstrating that metformin inhibits Hedgehog signaling via AMPK-dependent mechanisms and by interfering with GLI activity (33–36). However, the precise mechanisms in DLBCL remain to be elucidated. It is currently unclear whether metformin inhibits GLI transcriptional activity through AMPK-mediated signaling (37), or whether As_2_S_2_ promotes GLI2 ubiquitination, proteasomal degradation, or altered nuclear localization (38–40). Thus, although the present data support the involvement of Hedgehog signaling, the molecular sequence linking drug exposure to pathway suppression has not been fully defined.

The differential responses observed in DB and SU-DHL-4 cells further underscore the biological heterogeneity of DLBCL. SU-DHL-4 cells represent a germinal center B-cell-like (GCB) cell line, whereas DB cells exhibit characteristics more consistent with the activated B-cell-like (ABC) phenotype. Accumulating evidence indicates that metformin exhibits subtype-dependent activity in DLBCL, potentially modulating oxidative phosphorylation, glycolysis, and adaptive metabolic pathways in distinct manners (41). Previous studies have also demonstrated that metformin can synergize with targeted or metabolically active agents in a context-dependent manner, with efficacy varying across DLBCL subtypes (42, 43). Our findings are consistent with this framework and suggest that subtype-specific biological dependencies may influence both the magnitude and mechanism of the response to metformin-As_2_S_2_ combination therapy.

Beyond subtype-specific heterogeneity, the translational significance of the present findings should also be interpreted in light of the limitations of conventional two-dimensional monoculture systems, which do not recapitulate the complexity of the tumor microenvironment (TME). In DLBCL, therapeutic response is governed not only by tumor cell-intrinsic signaling but also by interactions among malignant B cells, immune cells, stromal components, soluble cytokines and other factors, and local metabolic constraints. Consequently, the synergistic effect observed in DB and SU-DHL-4 cells likely represents only one facet of drug activity and may be modulated in more physiologically relevant settings. Metformin is of particular interest in this regard, as its antitumor activity may extend beyond direct metabolic inhibition of lymphoma cells to include immunomodulatory effects within the TME, such as modulation of T-cell function and Treg-associated immune suppression (44, 45). Similarly, arsenical agents may exert broader biological effects beyond direct cytotoxicity, potentially influencing the surrounding cellular ecosystem; however, available evidence is derived primarily from studies of arsenic trioxide in other disease contexts and thus cannot be directly extrapolated to As_2_S_2_ in DLBCL (46). More broadly, accumulating evidence indicates that metabolic adaptation and inflammatory signaling are closely linked to the establishment of immunosuppressive microenvironments across various tumor types (47–51). Specifically, persistent activation of pathways such as IL-6/STAT3 has been associated with PD-L1 upregulation, polarization of tumor-associated macrophages toward immunosuppressive phenotypes, expansion of myeloid-derived suppressor cells, and impairment of CD8+ T-cell and NK-cell function, thereby reinforcing a self-sustaining inhibitory circuit within the TME (48, 49). Concurrently, emerging studies suggest that tumor-intrinsic metabolic programs, including those associated with ferroptosis resistance and redox adaptation, may actively contribute to immune evasion rather than merely accompanying it (50). Consistent with this concept, tumor cell-autonomous regulators such as ZHX2 and FBXL16 have been implicated in remodeling cytokine networks and immune cell states in a manner that favors local immune suppression (47, 51). Although much of this evidence originates from non-DLBCL contexts, these observations provide a biologically plausible rationale for evaluating the metformin-As_2_S_2_ combination in co-culture systems and *in vivo* models that incorporate relevant stromal and immune components.

Several limitations of this study should be acknowledged. First, all experiments were conducted using only two DLBCL cell lines under *in vitro* conditions, without validation in primary patient samples or *in vivo* models, thereby limiting physiological relevance. Second, the use of a restricted concentration range and a fixed treatment schedule precluded systematic evaluation of dose-response relationships, treatment sequencing, late apoptosis, and adaptive resistance. Third, our mechanistic analysis relied primarily on expression changes in apoptosis-related proteins and core Hedgehog pathway components, without functional interrogation of GLI activity or protein stability. The absence of reporter assays, ChIP-qPCR, cycloheximide chase experiments, MG132 rescue experiments, and ubiquitination analyses leaves the relative contributions of transcriptional regulation, proteasomal degradation, and post-translational control unresolved. Fourth, toxicity was not assessed in normal cells or animal models. Finally, because all experiments were performed in a standard glucose-rich culture system, it remains unclear whether the observed synergistic effect would be maintained under more physiological glucose conditions.

Future studies should proceed in several directions. Validation in a broader panel of DLBCL models, including additional molecular subtypes, primary patient samples, stromal co-culture systems, immune co-culture systems incorporating macrophages or T cells, three-dimensional spheroids, xenografts, and patient-derived xenografts, will be essential to establish robustness and translational relevance. Toxicity studies in normal cells and *in vivo* systems are also required. Mechanistically, genetic and pharmacological perturbation of SMO/GLI, combined with apoptosis assays and protein stability analyses, will be necessary to determine whether the combination treatment acts primarily through transcriptional repression, altered protein turnover, or both. In parallel, future studies should directly investigate TME-related endpoints, including tumor-associated macrophage polarization, CD8+ T-cell activation, NK cell function, Treg abundance, IL-13 dynamics, and broader cytokine network remodeling. It will also be important to compare simultaneous and sequential treatment strategies across multiple doses and time points. Finally, this combination treatment should be evaluated under lower, more physiological glucose conditions to determine whether the effects of metformin are context-dependent and whether the synergistic activity can be maintained at more clinically relevant exposures. Overall, our study provides initial evidence that metformin can potentiate the antitumor activity of As_2_S_2_ in DLBCL cells and identifies apoptosis-related signaling and Hedgehog pathway suppression as candidate mechanisms underlying this interaction. While these findings support further investigation, their translational significance will depend on validation in biologically more complex systems that better reflect the metabolic and immune context of DLBCL.

## 5 Conclusion

The present study demonstrates that metformin enhances the anti-proliferative and pro-apoptotic effects of As_2_S_2_ in DLBCL cells *in vitro*. These effects are associated with modulation of the BAX/BCL-2 axis and downregulation of Hedgehog pathway-related molecules. Although these findings warrant further investigation of this combination strategy, additional studies are required to establish causality, translational relevance, and safety.

## Data Availability

The original contributions presented in the study are included in the article/supplementary material. Further inquiries can be directed to the corresponding author/s.
